# Perceptions of overdose response hotlines and applications among rural and remote individuals who use drugs in Canada: a qualitative study

**DOI:** 10.24095/hpcdp.44.11/12.03

**Published:** 2024-11

**Authors:** Dylan Viste, William Rioux, Marguerite Medwid, Kienan Williams, Esther Tailfeathers, Amanda Lee, Farah Jafri, Stryder Zobell, S. Monty Ghosh

**Affiliations:** 1 Department of Medicine, Cumming School of Medicine, University of Calgary, Calgary, Alberta, Canada; 2 Department of Medicine, Faculty of Medicine & Dentistry, University of Alberta, Edmonton, Alberta, Canada; 3 Department of Nursing, Faculty of Health Sciences, University of Lethbridge, Lethbridge, Alberta, Canada; 4 Indigenous Wellness Core, Alberta Health Services, Calgary, Alberta, Canada; 5 Department of Family Medicine, Faculty of Medicine & Dentistry, University of Alberta, Edmonton, Alberta, Canada; 6 Alberta Health Services, Edmonton, Alberta, Canada

**Keywords:** overdose, drug poisoning, overdose response hotlines and applications, harm reduction, supervised consumption, public health, rural health, Indigenous health

## Abstract

**Introduction:**

The overdose epidemic continues to be one of the largest public health crises in Canada. Various harm reduction supports have been implemented to curb this epidemic; however, they remain concentrated within urban settings. To address this limitation, overdose response hotlines and applications (ORHA) are novel, technology-based harm reduction services that may reduce drug-related mortality for people who use substances (PWUS) living in rural communities through virtual supervised consumption. These services enable more timely and remote activation of emergency responses, should an individual become unresponsive. We aimed to explore the experiences, perceptions and attitudes surrounding ORHA of individuals living in rural areas.

**Methods:**

We conducted semistructured interviews with 15 PWUS (7 [46.7%] male, 9[60%] Indigenous) who lived in rural, remote or Indigenous communities. Interviews were conducted until data saturation was reached. Data were analyzed using thematic analysis.

**Results:**

Six key themes emerged: (1) participants viewed ORHA as a pragmatic intervention for rural areas but noted potential limitations to its uptake and effectiveness; (2) rural geography may hinder EMS response times, reducing the efficacy of ORHA; (3)ORHA uptake may be limited due to significant stigma faced by PWUS in these communities; (4) lack of access to technology remains a barrier to ORHA access; (5) harm reduction awareness is often limited in rural communities; and (6) there are unique social implications around substance use and harm reduction for rural Indigenous PWUS.

**Conclusion:**

While participants believed that ORHA may be a feasible harm reduction strategy for rural PWUS, limitations, including response times, technological access and substance use stigma, remain.

**Highlights**

- There are significant gaps in harm reduction services and awareness in rural areas.
- Significant stigma faced by people who use substances in rural communities drives additional caution in these individuals with respect to accessing harm reduction services, if they are even available.
- Overdose response hotlines and applications (ORHA) may be the only harm reduction services accessible to people who use substances in rural communities.
- Rural geography may pose challenges to emergency responses from ORHA services.
- Technology ownership and connectivity by people who use substances in rural communities may be limited, reducing service uptake.

## Introduction

The substance use mortality (also termed “overdose” or “drug poisoning”) epidemic crisis is arguably one of the largest public health issues currently facing North America.^1^ To combat the soaring mortality rate associated with this epidemic, various harm reduction strategies have been implemented across Canada which continue to prove effective at attenuating this crisis.^2^-^4^ Services such as drug-checking services, supervised consumption sites and risk mitigation guidance have resulted in reductions in morbidity and mortality rates.^5^-^7^

Access to these resources, however, remains a continuous challenge. As highlighted in a modelling study by Irvine et al., increasing uptake of harm reduction interventions, such as take-home naloxone kits, would likely lead to significant reductions in mortality rates from this epidemic.^8^ While the current literature remains mixed on the association between geospatial location and fatal overdose/drug poisoning,^9^,^10^ more recent data indicate that there is a 30% increase in the odds of fatal overdose within rural locations in British Columbia.^9^ The authors of the latter study hypothesize these differences originate most prominently from both a lack of harm reduction access and an increasingly toxic drug supply.^9^ Moreover, even within large urban centres, reductions in mortality attributed to supervised consumption services have only been documented within a 500-metre radius.^11^

Accordingly, people who use substances (PWUS) and policy makers have looked to novel strategies to increase the current reach of harm reduction in North America by leveraging the use of technology.^12^-^17^ Indeed, in an effort to keep communities of PWUS safe, the practice of virtual “spotting” was adopted: PWUS would call other members of the community or other trusted individuals from their social networks to witness their substance use session virtually and to activate an emergency response should the individual become unresponsive.^18^

Programs such as overdose response hotlines and applications (ORHA) aim to provide more timely responses to overdoses, particularly for those who do not currently access harm reduction services. ORHA programs provide remote interventions for overdose response that comprise both smartphone applications and telephone hotlines, and aim to decrease response times. Overdose response hotline services available in Canada include the National Overdose Response Service (NORS) and Brave app. In contrast, overdose response applications operate in select Canadian provinces, including the Digital Overdose Response Service (DORS) app and Lifeguard app, available in Alberta and British Columbia, respectively.^19^ In the United States, services similar to NORS exist, including the Never Use Alone and “SafeSpot” services. 

Due to the relative novelty of these services, there is currently a dearth of literature on their effectiveness; however, a study on one of the aforementioned services (NORS) provided early evidence of this service as a harm reduction strategy, with no reported fatalities across 3994 substance use sessions and 77 overdoses.^20^ Additionally, these interventions broadly have demonstrated a reduction in fatal overdose events and a favourable cost-benefit analysis.^19^,^21^ They have also served to support individuals with concurrent disorders, including methamphetamine psychosis.^22^ While these services have been beneficial for PWUS, one study of Canada’s National Overdose Response Service has found that uptake of this service is limited within rural communities (<1%).^20^

To determine how best to improve harm reduction access through ORHA, we set out to explore (1) the values, perceptions and beliefs among PWUS who live in rural, remote or Indigenous communities regarding the potential utility of ORHA in their communities; and (2) ways of improving the acceptability and effectiveness of ORHA to better meet the needs of PWUS in rural, remote or Indigenous communities. 

## Methods

***Ethics approval***

This qualitative study employed the COREQ guidelines for methods and results reporting,23 and ethics approval was obtained from the University of Alberta (Pro00118444). Participation was voluntary and verbal consent was obtained from all participants after a discussion about the implications and risks associated with the study. 

***Research team characteristics and reflexivity***

The core research team consisted of a research assistant (DV) and a student (MM), with two internal medicine residents (FJ and AL), as well as a specialist-trained physician (MG) with master’s level training in qualitative analysis. DV and MG had previous experience in conducting and evaluating qualitative studies and guided the trainees in qualitative methodology. Interviews were conducted by DV and MM, who also conducted the analysis thereafter. 

***Study design and research paradigm***

We employed a qualitative descriptive design for this study. We constructed a semistructured interview guide, informed by previous research conducted around rural harm reduction as well as professional knowledge and the experiences of PWUS. PWUS reviewed the content of the interview guide to ensure appropriateness and respectfulness. We used grounded theory and inductive reasoning to analyze the content of the interviews. Given that ORHA is a new technology with limited penetration, we chose this methodology because it allowed us to examine these novel technologies and evaluate their use and adaptation grounded in data.^24^,25

***Recruitment and sampling strategy***

We utilized purposive and snowball sampling between March and July 2023 to recruit and interview study participants. Sites of recruitment included rural harm reduction facilities and outreach programs. Additional participants were recruited from a large national harm reduction survey conducted by the research team (forthcoming), through addiction clinics and word of mouth. We approached participants through email or telephone. We focussed on recruiting participants from across Canada and from among individuals who lived in rural communities and small population centres with populations of less than 10 000. Participants were provided a $30 honorarium for their participation in the study. Participants were able to choose to have interviews conducted by a female (MM) or male (DV) interviewer, according to their preference. Eligibility and exclusion criteria are outlined below.

***Eligibility criteria***

To be included in the study, a participant must

- be a resident of a rural or Indigenous community or remote area across Canada;
- be aged 18 years or older;
- have reported use of unregulated substances (current or past);
- be able to communicate effectively in English and provide informed verbal consent;
- have a telephone number or email address; and
- have access to either a phone or a device with web chat features.

Participants were excluded if they

- were unable to speak English;
- were currently at risk of harming themselves; or
- required someone else to make decisions for them.

***Interview process***

After obtaining consent, interviews were conducted either via telephone or Zoom. All participants were informed that they could leave the interview anytime, for any reason, and remain eligible for an honorarium. A list of mental health and substance use support numbers was provided to support clients. A short survey was administered before the interview to obtain baseline sociodemographic information. Interviewers took field notes. Audio files were then transcribed by a third-party transcription service with identifying information redacted and stored on a secure and private hard drive at the University of Calgary. 

***Data analysis***

We used NVivo software version 12 (QSR International, Denver, CO, US) for the transcript coding process. All transcripts were coded by two independent members of the research team (DV and MM). The research team conjointly discussed all emerging nodes and themes to ensure alignment with coding and to analyze for saturation. Recruitment continued until saturation was reached, which was determined by a lack of new themes emerging across all participants. Member checking was conducted through comparisons with previous literature and a discussion of results with PWUS. Due to the large proportion of Indigenous participants within our study, the interview guide, study results and discussion were reviewed by two Indigenous partners (ET and KW) to ensure validity, cultural sensitivity and accurate interpretation of themes. 

## Results

Fifteen participants (mean age=38 years, SD=10.74; n=7 [46.7%] male) were recruited nationally. Nine (60.0%) identified as Indigenous and the remaining six as White (40.0%). On average, participants estimated that ambulances would take between 25:50 (19:53) and 31:40 (27:38) minutes to arrive at their homes in an emergency. Additional demographic and response data are outlined in Table 1. 

**Table 1:** Sociodemographic data of participants in ORHA study in rural and remote areas, Canada, 2023

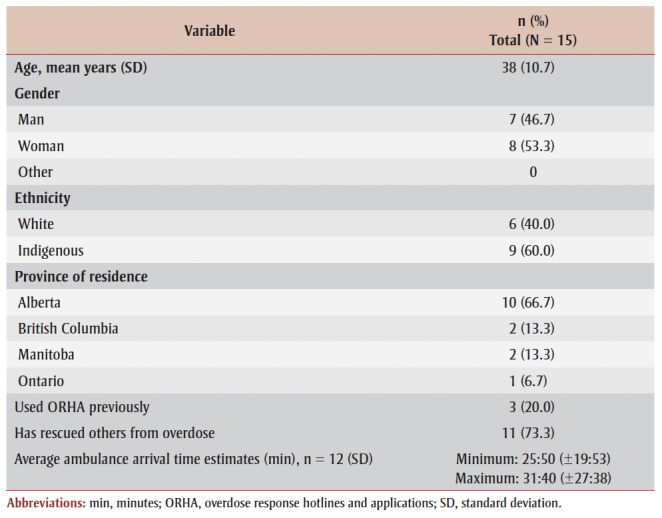

Six themes were identified from our thematic analysis, described below and summarized in Table 2.

**Table 2:** Major themes and key takeaway recommendations for ORHA engaging with rural, remote or Indigenous communities in Canada

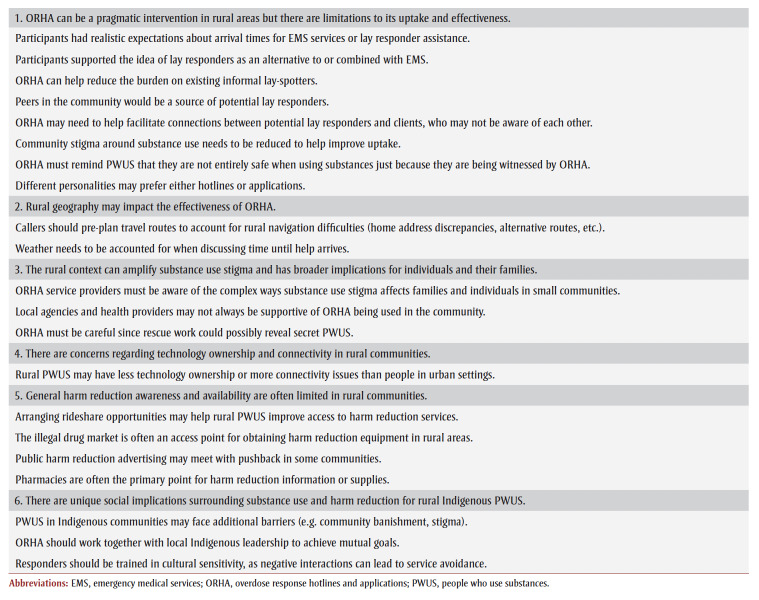

***Theme 1: ORHA can be a pragmatic intervention in rural areas but there are limitations to its uptake and effectiveness***

Within rural areas, participants felt that ORHA provided an additional (or possibly the only) harm reduction service that could potentially save lives. Despite concerns that the risk of delayed response could be harmful, it was deemed that this risk was much lower than that of using substances alone. Interviewees saw ORHA as a pragmatic solution, similar to other telecommunication or digital interventions employed to provide opportunities and services in rural communities. 

It’s like you use and you die, or you use and if you OD someone might come and rescue you. Which one would you rather have? No one’s coming, or someone might come? I feel like that would be a better option … (Participant #12, male) 

Many participants had previously engaged in informal spotting or used ORHA. There was unanimous support for using peers as service operators, which they felt added legitimacy to the program. Almost all supported having lay responders as an optional feature, as it could potentially be a faster alternative. However, they were still in favour of having emergency medical services (EMS) as a backup plan. Most participants said they would hypothetically be interested in being a lay responder for ORHA in their area. It was suggested that ORHA might need to help facilitate connecting known lay responders to PWUS, as finding a lay responder could potentially be stigmatizing or impossible for PWUS without personal connections. 

Participants who were currently receiving or providing spotting (virtually or in person) believed the use of ORHA would help reduce the burden on spotters, as volunteering to do this was viewed as disruptive of spotters’ personal lives.

And usually, for me, I usually call somebody or I’m messaging one of my friends on Messenger and I’m like, “Okay, I used today, and this is how much I used,” or “This is how much I’m going to use.” And they have their own lives, too, right? And they can’t just sit there and watch me on video chat. (Participant #14, Indigenous female) 

While engaging lay responders was mostly received positively, some interviewees did express that peers faced risks such as relapse during a response or burning out if they were constantly being the spotter for their community. Additionally, participants noted that they were not completely aware of others using substances in their community, making connecting to local spotters or using in a group less viable an option. 

Despite the support for ORHA, participants were less optimistic about the uptake of these services in their communities, often citing that those with the highest risk of overdose were less likely to use ORHA. They cited that key characteristics of these individuals included being too intoxicated to remember to use ORHA, a general lack of care for their own safety, or mistrust and stigma around harm reduction programs. 

I think it’s a great idea and it could help save a lot of people, but I know from the state of mind I was in during the worst time, I wouldn’t have cared. If that makes sense. I wouldn’t have cared if I was going to die. (Participant #4, female) 

Some participants were concerned that ORHA could lead to rural PWUS feeling a false sense of safety and they might begin using in riskier ways than when alone, unsupported by ORHA. 

That could definitely happen. Like, “Oh, I could use as much as I want when I want, I’m going to get saved.” (Participant #12, male) 

I feel like maybe if there’s a safeguard there, I would have pushed it farther. I feel like I might have. (Participant #7, female) 

Regarding which form of ORHA individuals would prefer to use, participants differed on whether they preferred hotlines or applications. In terms of hotlines, one participant mentioned: 

I think that’s actually fairly good because a lot of the time when they do use, they kind of go under, they start nodding, they’re not really aware of what’s going on in their surroundings. They’re more, I guess, nodding off, that’s how it is: falling asleep, going under. So I think that that would be a good idea because the loud noise might jolt them up out of it or if it’s been so long then the EMS is already on its way, you know what I mean? (Participant #12, male) 

Another participant preferred the applications, as they did not want to interact with others:

I think I would want to use the app, like, the one that beeped because it was like I would be by myself, even though it’s like the operator on the other line is somebody who is also a drug user or recovering addict…. When I was in my, like, deepest active addiction, the last thing I would want, well, felt like talking to, [making] small talk with strangers. You know what I mean? (Participant #7, female) 

***Theme 2: Rural geography may impact the effectiveness of ORHA***

The geographic isolation of rural communities was seen as a significant, but not insurmountable, barrier to the utilization of ORHA. Specifically, participants raised concerns about EMS arrival times to an overdose/drug poisoning event. Many rural participants had personal or second-hand experiences of longer EMS arrival times. 

Literally by the time 45 minutes is up you’re literally not going to make it, so that would be hard in the reserves. It would be really hard for an ambulance trying to find somebody living in the reserve. (Participant #3, Indigenous male) 

Besides physical distance and length of time to reach a destination, many participants stated that directing emergency help to their location would be difficult due to complexities such as EMS operators being unfamiliar with the terrain or rural road networks making it easy to get lost or miss the location. There were concerns about incorrect legal addresses (legal home addresses not matching the physical address on some houses). Some participants did not know how to easily give their locations, relying on landmark-based navigation instead of their official street address. It was suggested that ORHA consider discussing the entire travel route with rural PWUS, not just the end location, to minimize the aforementioned complications.

So, yeah, I just wanted to get them there. They’re like didn’t want them to get lost and like we’re here, hurry up, we’re here, just because some of the houses do not have numbers on them. I don’t know why. (Participant #9, Indigenous female) 

Adverse weather events were also listed as being a barrier to effective EMS arrival times. Even though these events were uncommon, the effects of adverse weather are often magnified in a rural setting. Adverse weather could potentially stop communication networks or worsen driving conditions, both of which would impact the effectiveness of ORHA. 

Trees do come down, roads get blocked, so that could pose a problem. (Participant #11, Indigenous male) 

***Theme 3: The rural context can amplify substance use stigma and has broader implications for individuals and their families***

Participants identified that rural communities were unique in that “everyone knows what everyone is doing,” and this interplay between personal reputation in small communities and substance use stigma was often (but not always) listed as a major concern. The consequence of being a known substance user was described as an immediate decrease in social standing and reputation. This was experienced by various participants or was witnessed through the treatment of other people who use substances in their community. 

It might help a little bit just to know that they’re not going to get in trouble because that’s probably, the biggest thing for an addict—is people finding out. (Participant #3, Indigenous male) 

Substance use was identified not only as affecting the reputation of the person who uses substances but also as a concern for one’s entire family. Family members often distance themselves from people who use substances, or attempt to conceal the person’s use. The fear of impacting an entire family’s reputation was especially important, as most participants described themselves as being particularly close to their families.

They [families] don’t want to … they might not even tell their other family members that they have a son that’s messed up. They might not tell their brothers and sisters or even their mother or grandparents or whatever. They kind of keep it a secret because they feel ashamed, I guess.… (Participant #3, Indigenous male) 

A lot of people are not able to be open and honest with their family. And then when they do, when family does find out about people using, I notice they get put into a stereotype or they get shunned, especially with meth, I noticed a lot of families push away their loved ones that are using. (Participant #14, Indigenous female) 

Almost all participants (with the exception of two) expressed that their primary reason for staying in their community was their family. Even when they did temporarily leave, they continued to gravitate towards their home community because of family. 

While generalized substance use stigma within rural communities remains a concern, participants did not feel as though rural community members would actively protest against ORHA. 

The townspeople could get a little pissy, I guess, I don’t know. They are, well, I guess it’s virtual. It’s like on your phone itself, it’s not like a safe injection site but like ... I don’t know. I don’t think there would be any negative to it really. (Participant #12, male) 

While ORHA were thought to be life-saving, occasionally they were described as stigmatizing, but no more so than any other harm reduction services. Stigma-based obstacles preventing ORHA included health care or social service providers who are not sympathetic to PWUS and could therefore deter patients from accessing services where they could be introduced to ORHA. Additionally, participants expressed the fear of being revealed as a PWUS by the arrival of EMS or rescuers to their home. 

Because if you see an ambulance going down the street, everybody stops and stares. Whose house are they going to? What happened there? And why is this person doing that? So nosey, it’s unbelievable. (Participant #4, female) 

***Theme 4: There are concerns regarding technology ownership and connectivity in rural communities***

Participant perspectives were mixed regarding access to technology, cellular reception or data and phone minutes needed to access ORHA in rural communities. Participants were split on whether rural PWUS were more or less likely to have technology compared to those in urban settings. Specifically, there existed a fear that technology was too easy to sell in exchange for substances, leading to situations in which the technology needed for improving safety would be absent when people were at their most vulnerable. 

I think if somebody can afford a phone, they can have a phone and keep a phone without selling it for drugs, I think it would be a really good thing. That’s another thing, too. Do they have minutes? Can they call anybody? (Participant #9, Indigenous female) 

In addition, a few participants raised concerns about data privacy and data protection while using the various ORHA. 

***Theme 5: General harm reduction awareness and availability are often limited in rural communities***

Awareness of and access to harm reduction resources in rural communities was highlighted as being particularly challenging. One of the most frequently recurring barriers was the difficulty of obtaining transportation to certain harm reduction supports, particularly due to the absence of any public transit or professional driving services. This lack led to the necessity of relying upon community members or family rideshares, which rarely were offered freely or in a timely manner. Some participants were already offering free rides for those beginning their addiction recovery, as they had personally experienced or observed the difficulty of making it to harm reduction service locations or attending recovery-oriented appointments. 

Like, it was so impossible. Like, I tried going to the hospital. I tried going to mental health services. And, like, all they said was, you got to go to Grand Prairie to this place. So I knew this magical place in Grand Prairie existed, I just didn’t have any way to get there. (Participant #7, female) 

Participants usually felt that they had fewer harm reduction service options than in urban areas. The most commonly listed harm reduction services were pharmacies providing sterile equipment or replacement medication treatment, followed by small community health centres containing minimal addiction resources. Some of the communities were said to have no resources at all or participants were unfamiliar with any resources in their area. Participants also highlighted that their personal harm reduction supplies often came from out of town via other mobile supply services, or they were provided conjointly with drug acquisition. Drug dealers would often provide harm-reduction supplies while selling substances. Other participants stated they would often stock up on harm-reduction supplies while visiting a larger community. ORHA as a harm reduction tool was seen as being a reasonable adjunctive option to ensure safety. 

Though neither stigma nor ideology in smaller communities was seen as a barrier to implementing or using ORHA, stigma was still seen as a barrier in increasing general awareness of the service: 

Well, I wish I had more info I could hang up in the community, but I don’t know how the people would take it. It’s—some people would accept it, and then there’s other people are, like, “What? Why are you bringing that kind of negativity?” ... Like, it’s not negativity—I think it’s just trying to help people from OD’ing, but ... our community is always divided. (Participant #5, Indigenous male) 

I know that being in a small community there is this huge stigma around that, say you needed Narcan and clean needles, to go into the hospital and ask for that there. I’ve heard that it’s a very tough situation because a lot of the nurses over there will look at the person and just be like, “Well, you know, there’s that person.” (Participant #4, female) 

Additionally, participants felt that town or community approval might be required to disseminate advertisements on controversial topics such as harm reduction, which could further limit awareness of these services. It was felt that advertising harm reduction in some communities was essentially admitting that there was a problem in their community, which makes the community “look bad.” Despite this, several suggestions were made regarding increasing general awareness of the service: 

I think I’m going to make a flyer and hang it in my community centre … right at the door. They got a big bulletin board. They hang everything. I put one there—I put one all over so they could see it, because if I could save a life, that’d be great just from … them copying that [NORS hotline] number. (Participant #5, Indigenous male) 

Social media was another outlet seen as being reasonable for helping to create awareness of the service, especially as the participants believed that it would require less authorization and approval from local authorities. Many participants noted that the smaller communities did not have their own dedicated web pages and instead used the pages of nearby, larger communities to share information. 

The town of [redacted] has a [Facebook] page where they post things going on around, right? Stuff like that. (Participant #7, female) 

***Theme 6: There are unique social implications surrounding substance use and harm reduction for rural Indigenous PWUS***

Over half of our participants identified as Indigenous or being from Indigenous communities. Most of the Indigenous participants shared experiences similar to those of non-Indigenous participants, such as transportation barriers and lack of harm reduction resources in town or nearby. Indigenous participants highlighted experiences of community stigma and the effectiveness of lay responses, impacted in part by their Indigenous culture, kinship and community bylaws.

All participants shared that substance use was often seen as a failure, and was frequently hidden due to shame, fear or other internalized negative feelings. Indigenous participants expressed pressure to conceal substance use to avoid feeling shame in their community, the consequences of which could impact obtaining community leadership positions such as chief and council positions. Participants referred to bylaws entrenched and enforced in some Indigenous communities that evict community members who are deemed to be contributing to the substance use problem, which can lead to cycles of substance use and increased movement between multiple communities. 

A lot of them get kicked out, and then they live on the streets in [small town], and then claim to get clean, and then they come back into the house, start stealing again, get kicked out. (Participant #5, Indigenous male) 

The fear of one’s home being labelled as a house where substance abuse occurs is a barrier to establishing a network of in-community lay responders; their vehicle in the driveway may be noticed by family or community members. The professional and societal consequences of the stigma may outweigh the benefits of ORHA. Additionally, establishing a lay response network without the inclusion of Indigenous community leadership could have negative implications, especially if they are supporting previously evicted community members. 

I would think, you know, that should be an actual thing where people with actual experience and knowledge in saving people in this aspect has really benefits. Like, so many people now that have OD’d since my [female relative 2] and I have been evicted and banished from our home reserve. There’s been so many deaths there. (Participant #9, Indigenous female) 

Although it did not reach thematic saturation, participants expressed concern about racism compounding stigma, which could lead to decreased adoption of ORHA: 

I’ve heard it all. I’ve been in ambulances where they think I’m unconscious and they’re talking about the “Indians” of [small town] and how it’s so annoying to drive way out there to help somebody. Yeah. I know it has to do with the colour of my skin. (Participant #5, Indigenous male) 

## Discussion

Our evaluation demonstrates a complex interplay between the need for harm reduction resources such as ORHA in rural communities, and the difficulties in implementing them and increasing awareness and use of the program due to geographic practicalities, stigma, technology infrastructure and cultural complexities. Several key messages emerged, with implications for program implementation and public health policy. 

All participants acknowledged the general barriers to obtaining harm reduction support in rural communities, highlighting transportation issues and lack of service provision as two prominent concerns. Previous literature has highlighted these same barriers and demonstrated an increased risk of paraphernalia sharing, paraphernalia reuse and use of substances while alone.^26^ While ORHA may not yet mitigate concerns about risky paraphernalia use, they might help reduce risks associated with using substances alone. 

Nonetheless, concerns about EMS arrival times, stigma from EMS services, and rescue services getting lost while being dispatched have been seen in the literature. One retrospective study conducted across the United States showed that emergency medical service response times in rural areas are nearly double those seen in larger urban settings.^27^ Awareness of the limitations of EMS response in rural communities should be appropriately communicated to PWUS using ORHA, and efforts to reduce delays in overdose response in these communities should be made. Though establishing community-based lay responders has been shown to be a viable strategy,^28^ it is also seen as challenging to implement in rural and remote communities due to fears of burnout, lack of community-based responders or relapse among peer responders. 

Participants expressed some concerns around data collection and surveillance from ORHA. Within the context of rural communities, people are often more mistrusting of the government, and therefore may not want to use government-sponsored services.^29^ In Canada, various ORHA have different operation and funding models, with some providing direct operational support and funding, some providing only government funding and some being privately funded and operated.^19^,^30^

The importance of family was a unique theme not seen before in previous literature around rural harm reduction. Family not only motivated PWUS to stay in their communities but also impacted their willingness to seek support for their substance use, whether harm reduction or treatment, due to the risk of stigma and harm to their family’s reputation. All participants shared the theme of bringing shame to one’s family or community; however, the consequences and impact differed between Indigenous and non-Indigenous communities. Recognizing that each community faces unique challenges to developing and managing substance use can lead to increased program success, suggesting the need for further education around substance use with a stronger focus on destigmatization appropriate for the community of residence. 

Previous work has highlighted the loss of social capital and the stigma associated with rural substance use in pregnant women, ethnic minorities and rural environments in general.^31^-^35^ Because many of the participants reported a family member as their primary designated lay responder (e.g. “My dad is just upstairs and checks in on me”), we believe that the family of PWUS will likely be a primary source of sympathetic and competent community lay responders, which could help return social capital to those disenfranchised by other stigmatizing forces. 

Access to harm reduction services in rural areas is limited in Canada and the US.^36^,^37^ The current distribution patterns of harm reduction supplies in rural communities (through official channels such as pharmacies and unofficial channels such as dealers and peer community sharing) were discussed and could be a vehicle for disseminating information on ORHA. Posters and social media were also considered reasonable strategies to disseminate information regarding these strategies; however, this messaging may not be permitted within communities with particularly stigmatizing attitudes towards substance use. Because almost all of the participants either had a naloxone kit, planned to obtain one or helped distribute them to others in their community, the use of stickers or other promotional materials within naloxone kits could potentially promote or remind PWUS to use ORHA.^38^

Both interpersonal and structural racism and discrimination were additional layers of stigma highlighted by Indigenous participants, which is aligned with other research.^39^ Interpersonal racist attitudes towards racialized people, including Indigenous PWUS, are well documented in the literature,^40^ and the development of substance use disorders is linked to racism.^41^,^42^ Furthermore, Indigenous interviewees indicated that racism was a barrier to the use of ORHA, a finding consistent with previous work that has shown that racism is a barrier to calling 911 in overdose situations.^43^ Antiracist approaches are needed at all levels, including among first responders, to address these barriers.^44^,^45^

While many other instances of structural or institutional discrimination were discussed by participants (such as lack of community economic development or infrastructure improvements to road systems) and have been noted in the Canadian literature,^46^ they are beyond the scope of this paper. Our study highlighted how in Indigenous communities there are sometimes discrepancies between the legal and physical home address, which can cost precious minutes during an emergency response. Seen through an advocacy lens, it would be prudent to improve addressing in these areas to reach national parity. 

Our study highlighted participant concerns regarding the ability of emergency medical services to locate clients within a reasonable time frame. While one of the most immediate concerns discussed by participants was giving EMS more specific information about how to reach a client, another option would be for EMS to use ambulance GPS technology in concert with smartphone technology. GPS units have been shown to significantly reduce EMS response times for motor vehicle collisions, which usually occur on a roadway, by one minute.^47^ Some ORHA are directly connected to provincial EMS and enable sharing of GPS coordinates; future studies should examine whether these services may enable more rapid responses to individuals who have suffered an overdose event. 

Another participant suggested that building a network of spotters and responders could enable greater connections and more peer-based support within smaller communities, possibly improving the community’s overall wellness. While lay responders would likely benefit a community, it is imperative to consider the legal implications of reliance on lay responders under the *Good Samaritan Drug Overdose Act*^48^ in order to protect both harm reduction services and the lay responders. In Indigenous communities, ORHA will likely need to consider community bylaws when connecting lay responders to avoid legal or cultural conflict arising from both assisting or being assisted by a person who is currently banished from the community.^49^-^51^

Another important aspect of ORHA highlighted by participants is the potential risk for PWUS to increase substance use while using these harm reduction services, due to perceptions of decreased risk associated with their use. Previous studies have shown that PWUS tend to underestimate the risk of overdose.^52^,^53^ In contrast, studies of in-person supervised consumption sites note that there were no increases in harmful substance use in conjunction with using these harm reduction facilities.^54^ This makes it difficult to conclude the effectiveness of ORHA for rural populations. Future studies should examine any potential changes in substance use patterns, both pre- and post-service use, in addition to any potential increased risk of overdose mortality in rural settings due to delayed response times. 

Access to technology was presented as a barrier to the operation of ORHA in rural settings. Previous research on PWUS in downtown Vancouver noted that only 45% of individuals accessing supervised consumption services have access to mobile phones.^55^ While this number may not represent PWUS in rural settings, it is important to note that the digital divide likely persists within these communities. Reducing the digital divide for these communities may not only help to reduce barriers to harm reduction through ORHA but also through other internet-based services, such as mail-order harm reduction programs,^56^,^57^ social supports and treatment programs such as Alberta’s virtual opioid dependency program.^56^

***Strengths and limitations***

Our study has several main strengths, including furthering knowledge of substance use and attitudes towards harm reduction in rural, remote and Indigenous communities in Canada. The results, however, should be interpreted in the context of a few limitations. While we focussed on a variety of rural PWUS, it should be noted that there was great heterogeneity among the types of rural communities within the study sample, and thus our results may not be generalizable to every community.^58^ All respondents in our study had access to cell phones and technology, so their responses would represent a subpopulation of rural PWUS. Our recruitment methods tended to favour individuals already accessing harm reduction resources or treatment services, and we did not engage with individuals who did not have access to any supports. Many of the respondents were from British Columbia and Alberta, and their perspectives may not be generalizable to the rest of Canada. 

## Conclusion

The interviewed members of rural, remote and Indigenous communities suggested that ORHA could be a lifesaving and socially appropriate harm reduction resource, particularly as substance use stigma was perceived to be more intense in these communities. Most participants viewed ORHA as being safer than using substances alone and were hopeful that a combination of both EMS and in-community layperson rescues could save lives. ORHA should adopt the following features in rural settings: training in understanding rural addressing and rural (often informal) navigational strategies; factoring in adverse weather when describing potential wait times; and working to establish lay-responder allies in communities (with proper mental health support and legal protection) to help mitigate the longer EMS arrival times while at the same time striving to provide anonymous and discreet services that protect the privacy of PWUS. Lastly, technology ownership and cellular connectivity were highlighted as continued barriers to access for PWUS within these communities. 

## Acknowledgements

We would like to sincerely thank the participants for their time in participating in this study. We would like to thank Reed Charbonneau and Victoria Horn for their assistance in recruitment. We would also like to thank Dr. Tyler Marshall for providing methodological support, and Adrian Teare and Jayelle Warken for their support during the data collection of this project. 

## Funding

This study was funded by a contribution from Health Canada’s Substance Use and Addictions Program (SUAP). This study was also funded by Canadian Institutes of Health Research (CIHR). The study design, data collection and analysis, interpretation of results, and decision to submit for publication were done independently of SUAP and CIHR.

## Conflicts of interest

MG co-founded the National Overdose Response Service (NORS), and belongs to the Canadian Society of Addiction Medicine and has no personal financial conflicts of interest to disclose. The results of this work may be used to apply for funding for NORS or to make operational changes to NORS. The rest of the authors are unaffiliated with NORS in particular or any other ORHA, and have no competing interests to declare. 

## Authors’ contributions and statement

DV, MG: conceptualization.

DV, MM, AL, FJ: data curation.

DV, WR, MM, AL, FJ: formal analysis. 

MG: funding acquisition.

DV, MM, AL, FJ: investigation.

DV, MM, AL, FJ, SZ, MG: methodology.

MG: supervision.

DV, WR, MG: writing—original draft.

DV, WR, MM, KW, ET, AL, FJ, SZ, MG: writing—review and editing.

The content and views expressed in this article are those of the authors and do not necessarily reflect those of Health Canada or of the Government of Canada.
